# Valuing informal carers’ quality of life using best-worst scaling—Finnish preference weights for the Adult ﻿Social Care Outcomes Toolkit for carers (ASCOT-Carer)

**DOI:** 10.1007/s10198-021-01356-3

**Published:** 2021-09-01

**Authors:** Lien Nguyen, Hanna Jokimäki, Ismo Linnosmaa, Eirini-Christina Saloniki, Laurie Batchelder, Juliette Malley, Hui Lu, Peter Burge, Birgit Trukeschitz, Julien Forder

**Affiliations:** 1 Finnish Institute for Health and Welfare (THL), Helsinki, Finland; 2 Department of Health and Social Management, University of Eastern Finland, Kuopio, Finland; 3 Centre for Health Services Studies (CHSS), University of Kent, Kent, UK; 4 Personal Social Services Research Unit (PSSRU), University of Kent, Kent, UK; 5 Care Policy and Evaluation Centre, London School of Economics and Political Science, London, UK; 6 RAND Europe, Cambridge, UK; 7 Research Institute for Economics of Aging, WU Vienna University of Economics and Business, Vienna, Austria

**Keywords:** Adult Social Care Outcomes Toolkit for carers (ASCOT-Carer), Informal care, Outcome measurement, Quality of life, Evaluation, Best-worst scaling (BWS), Scale multinomial logit, Learning and fatigue effects, C35, C90, I18, I31, I39

## Abstract

This study developed Finnish preference weights for the seven-attribute Adult Social Care Outcomes Toolkit for carers (ASCOT-Carer) and investigated survey fatigue and learning in best-worst scaling (BWS) experiments. An online survey that included a BWS experiment using the ASCOT-Carer was completed by a sample from the general population in Finland. A block of eight BWS profiles describing different states from the ASCOT-Carer were randomly assigned to each respondent, who consecutively made four choices (best, worst, second best and second worst) per profile. The analysis panel data had 32,160 choices made by 1005 respondents. A scale multinomial logit (S-MNL) model was used to estimate preference weights for 28 ASCOT-Carer attribute levels. Fatigue and learning effects were examined as scale heterogeneity. Several specifications of the generalised MNL model were employed to ensure the stability of the preference estimates. The most and least-valued states were the top and bottom levels of the control over daily life attribute. The preference weights were not on a cardinal scale. We observed the position effect of the attributes on preferences associated with the best or second-best choices. A learning effect was found. The established preference weights can be used in evaluations of the effects of long-term care services and interventions on the quality of life of service users and caregivers. The learning effect implies a need to develop study designs that ensure equal consideration to all profiles (choice tasks) in a sequential choice experiment.

**Supplementary Information:**

The online version contains supplementary material available at 10.1007/s10198-021-01356-3.

## Introduction

The provision of long-term care (LTC) for older people in various OECD countries has been substantially contributed to by informal carers [1]. The projected growth in the LTC needs in Europe has imposed a difficult question of how to effectively allocate limited resources within LTC systems to support people with LTC needs and their informal carers [2, 3]. Concerning the supply side of informal care, providing care can lead to unfavourable effects on carers’ health, well-being, life satisfaction and overall quality of life (QoL). High-intensity caregiving is found to be associated with worse mental health, increased emotional and physical strain, and loneliness or feelings of isolation [4–7]. It is also associated with decreased life satisfaction [8] and increased use of drugs and outpatient care [9].

Systematic reviews [10, 11] indicate that informal carers’ well-being, stress or burden, mental health, needs and experience have been measured by a number of measures, such as the Caregiver Burden Interview [12], the CES Depression Scale [13] and the Social Satisfaction Scale [14]. Since these measures focus on specific aspects of carers’ well-being, they may omit outcomes or experiences that are important to carers. The use of appropriate measures and methods to assess the costs and outcomes related to the provision of informal care and the QoL of carers has become particularly important in effectiveness and cost-effectiveness studies that include informal care [15].

Adult social care aims to promote the well-being and QoL of adults needing support with daily activities and their informal carers (caregivers). The Adult Social Care Outcomes Toolkit for service users (ASCOT) was developed to measure adult care recipients’ social care-related quality of life (SCRQoL) and the effectiveness of support and services [16]. As carers’ outcomes and experiences differ from those of services users, the Adult Social Care Outcomes Toolkit for carers (ASCOT-Carer) was also developed [17, 18], and English preference weights for the original measure were recently derived [19]. The ASCOT-Carer can be used in effectiveness and cost-effectiveness evaluations of interventions focusing on social care and support to caregivers [18].

Similar to numerous generic preference-based measures [20], the English ASCOT-Carer preference weights [19] capture the values of the general population for ASCOT-QoL states in England. This reflects the point of view that the values of the general population should be used in decisions about how to allocate the limited resources in the health and social care sector as the general population pays for services and the provision of services is tax-funded in many European countries [21]. Furthermore, comparative studies have indicated that the general population’s preferences differ between countries according to culture and health and social care delivery systems [20, 22, 23]. Therefore, we should be cautious about valuing QoL states in one country using preference weights for QoL states that were developed in the context of another country [22, 23]. In the field of health-related QoL measurement, the usual practice is to develop country-specific preference weights to better explain the country’s own populations’ perceptions and values regarding various health states [24–26]. This approach was taken for translated-versions of ASCOT [27–29] (in German, Japanese and Finnish) and ASCOT-Carer [30] (in German ) measures.

To apply the ASCOT-Carer measure in Finland, we translated the measure from English to Finnish in 2015–2016, following international guidelines in the translation process [31].^1^ Since the preference weights for the Finnish-translated measure has not been developed yet, the primary aim of this study was to estimate Finnish preference weights for the Finnish ASCOT-Carer measure. Following Netten et al. [16], we collected choice data from a web-based general population survey that included a best-worst scaling (BWS) experiment [32, 33]. Using the BWS data and multinomial logit models, we estimated the preference weights for attribute levels of the Finnish ASCOT-Carer.

The recent literature on choice experiments has indicated that sequential choice tasks can give rise to learning or fatigue [34–36], where respondent choices become more consistent (learning) or less consistent (fatigue) over the course of the experiment. In the BWS experiment, each respondent had eight sequential choice tasks and made four consecutive choices per task. Since these repeated tasks created a prerequisite to explore fatigue and learning during the choice experiment, an auxiliary aim of the study was to investigate the effect of learning and fatigue on respondent choices and preference estimates in the BWS experiment. This study contributes to enlarging the number of valid measures for use to evaluate capability-based QoL in a general population [37] and better understanding the effect of fatigue and learning on respondent choices in BWS experimental studies.

## Methods

### ASCOT-Carer, best-worst scaling (BWS) and BWS tasks

The ASCOT-Carer measure has seven four-level attributes concerning different aspects of informal carers’ SCRQoL: (1) occupation; (2) control over daily life [control]; (3) looking after yourself [self-care]; (4) personal safety [safety]; (5) social participation and involvement [participation]; (6) space and time to be yourself [space-and-time]; and (7) feeling supported and encouraged [support] (Table 1). The attribute levels measure carers’ need intensity: Level_1 (top level) indicates the most favourable situation—the ‘ideal state’—and level_4 (bottom level) indicates the least favourable situation, i.e. ‘high needs’, whereas level_2 and level_3 indicate in-between situations (i.e. ‘no needs’ and ‘some needs’, respectively).

**Table 1:** ASCOT-Carer attributes describing informal carers’ social care-related quality of life

Attribute	Definition
Occupation	Being sufficiently occupied in a range of meaningful, enjoyable activities, whether it be formal employment, unpaid work, caring for others or leisure activities
Control over daily life	Choosing what to do and when to do it and having control over one’s daily life and activities
Looking after yourself	Feeling able to look after oneself in terms of eating well and getting enough sleep
Personal safety	Feeling safe and secure, where concerns about safety can include fear of abuse or other physical harm or accidents that may arise as a result of caring
Social participation	Being content with their social situation, where the social situation includes sustenance of meaningful relationships with friends and family, as well as feeling involved and part of their community
Space and time to be yourself	Having space and time in everyday life. Enough time away from caring to have a life of their own outside of the caring role
Feeling supported and encouraged	Feeling encouraged and supported by professionals, care workers and others in their role as a carer

Source. Rand et al. [18]

**Fig. 1:**
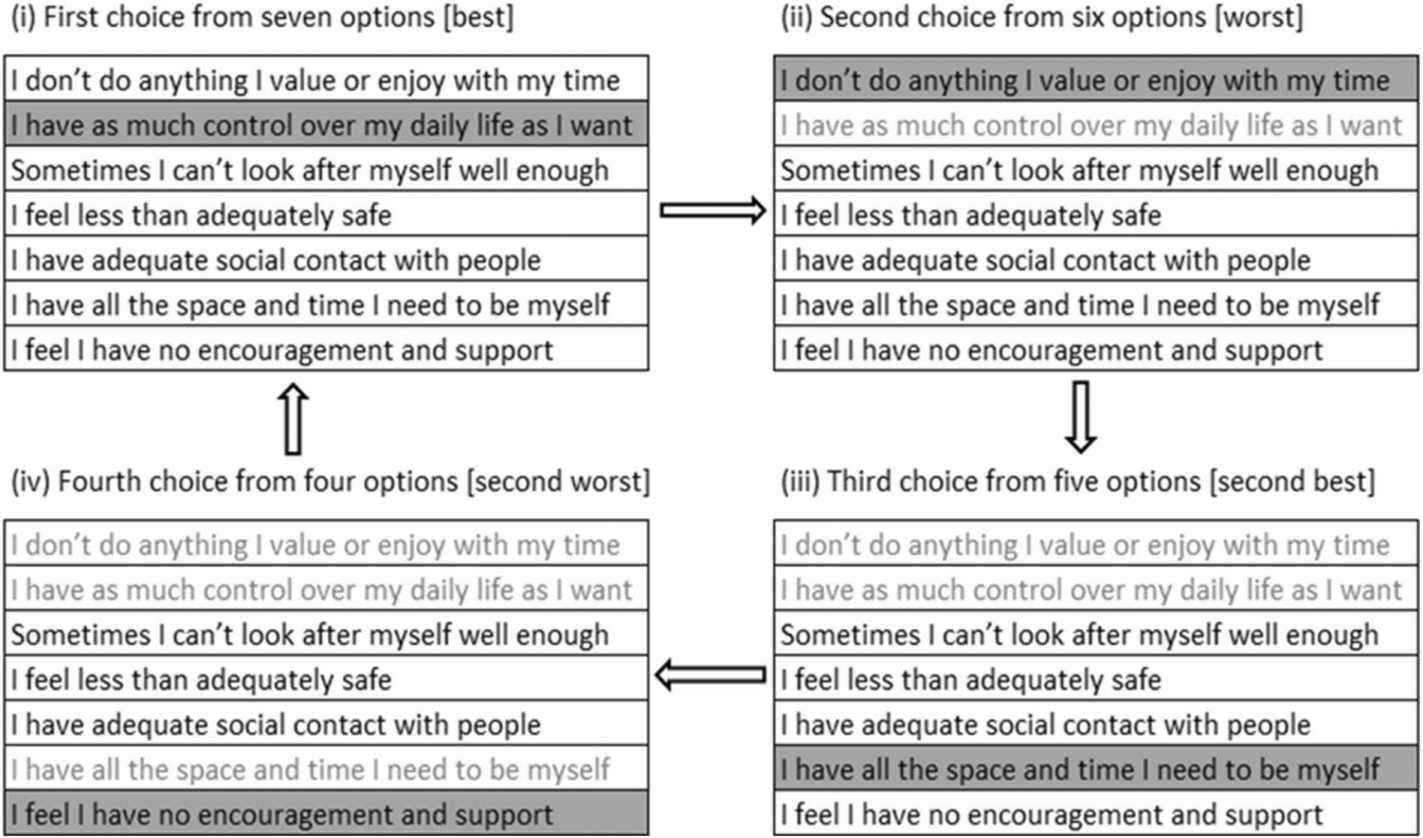
An example of a BWS profile using different QoL states from the ASCOT-Carer measure. ©University of Kent: The ASCOT-Carer measure is reproduced with permission from the University of Kent. All rights reserved

Following the approach used in Netten et al. [16], we used the BWS method to collect data to derive Finnish preference weights for the Finnish version of the ASCOT-Carer measure. The choice of the method used in [16] was informed by results from previous reviews [38, 39] which suggest that more information within choice sets can be obtained with less cognitive burden using the BWS method than using the DCE method. In the BWS profile case, one profile is presented at a time, and choices between alternatives are made within the displayed profile [40]. To reduce the effects of lexicographic and non-trading behaviour in the BWS tasks [41] and to obtain partial ranking for the attribute levels [39], the second-best and second-worst attribute levels from each profile were also selected (Fig. 1).

The full factorial design plan comprised 4^7^ possible profiles [38, 39]. To obtain a reasonable number of possible profiles to be presented to respondents, a fractional-factorial orthogonal main effects plan (OMEP) design of 32 profiles was used [42, 43]. Each profile included seven attribute levels, one from each attribute defined in the ASCOT-Carer measure (Fig. 1). The profiles were randomly divided into four blocks of eight profiles. Each respondent randomly received an eight-profile block. Respondents first imagined a situation where they would have cared for a person who needed help in their daily lives due to old age, disability or illness. Then, they evaluated the alternatives in the profile and sequentially selected four alternatives that gave the greatest and lowest relative utilities, making a BWS choice task. The number of alternatives available per profile decreased after each choice and the best, worst, second-best and second-worst choices was sequentially made per profile in each BWS task before moving to the next profile and a new task (Fig. 1).

A foldover design was applied to reduce the number of easy choices from each profile [44]. To reduce selection bias, the blocked profiles were randomly assigned to respondents. The position (order) of attributes was randomised between (but not within) respondents to avoid ordering bias and disengage the effect of attribute choice from the position of that attribute within a choice task [16, 35, 45].

### Survey design and sampling

An online survey that included the BWS experiment using the ASCOT-Carer measure was conducted between July and August 2016 and managed by Research Now. To achieve a representative sample of the Finnish general adult population for this survey, an online panel with quota sampling by age, gender and region was used. Besides the BWS choice data, we also collected information about respondents’ demographic and socioeconomic background (such as gender, age, region, household income, education, marital status, religion, employment status and tenure), well-being (self-assessed health (SAH) and overall QoL), information on experience in caring and need for social care as well as information about how well the respondents understood the given BWS tasks.

Those who spent less than 4.5 min completing the BWS task section were excluded during the data collection. At a testing phase, we found that it took at least that amount of time to read and complete eight BWS tasks (32 choices). Due to power calculation requirements, we continued sampling until the target of 1000 participants was reached, but we obtained a slightly larger sample at the end of the data collection (*n* = 1009). Excluding those with no information on education (*n* = 4), the final sample consisted of 1005 respondents, and the long-format panel data had 32,160 choices.

### Modelling strategy

The BWS choices were analysed based on the random utility theory [33, 46]. The estimated preference parameters are a function of choice frequencies, and the choice of an attribute level describes the importance of that attribute level relative to other available attribute levels [40]. To start out estimating the coefficients of the attribute levels, we first applied a multinomial logit (MNL) model. As existing scale heterogeneity capturing the variance of the error term in a random utility model can distort preference estimates obtained from the MNL model [47], to account for differences in different subgroups’ error variances and obtain more reliable and consistent preference estimates, we used a scale MNL (S-MNL) model [16, 38, 48] (Table 2).

**Table 2:** Model developing process and specifications

Estimation step		Model	Variable specification	Result
1	Basic model	MNL	Attribute levels and position variables of the attributes (for the best or second-best choices, and for the worst and second-worst choices) were included to the model	Model [I] (Table 5)
2	Taste model	Mixed logit	We included to the basic model (step 1): (a) the attribute-specific constants (ASCs) for the worst or second-worst choices, and (b) the interactions between the individual characteristics (e.g. age, gender, education) and the attribute levels to explain taste heterogeneity. We aimed to control for taste heterogeneity and minimise unexplained variations	*Not reported*
3	Taste-and-scale model	G-MNL	We included to the taste model (step 2): different sets of 4–5 variables at a time to investigate whether these variables could account for scale heterogeneity	*Not reported*
4	Scale model	S-MNL	We kept the significant scale factors obtained from step 3 and the position variables. We excluded the ASCs for the worst or second-worst choices and the taste variables explaining taste heterogeneity	Model [II] (Table 5)
5	Taste-adjusted scale model	S-MNL with taste variables	We included to the scale model (step 4) several significant interaction terms (taste variables) to adjust taste differences between the sample and general populations caused by the unrepresentative sampling	Model [III] (Supplemental Table S1)
			Using results from Model [III], we derived final population-based preference weights	Model [III*] (Table 5)

To select appropriate scale factors for the S-MNL model, we sequentially estimated two specifications of the generalised MNL (G-MNL) model [48] before estimating the S-MNL model. The first model used observed respondent characteristics to investigate taste heterogeneity (hereafter, *taste MNL* model). This was the MNL model expanded with (i) the attribute-specific constants (ASCs) for the worst or second-worst choices and (ii) interaction terms between attribute levels and observed characteristics of respondents to control for the variation in preferences for attribute levels between the subgroups of respondents. The second model, G-MNL, allowed for both taste heterogeneity and scale heterogeneity (hereafter, *taste-and-scale MNL* model). Hence, after having controlled for taste heterogeneity and minimised the unexplained variation of the model, we explored heterogeneity related to the error variance and selected the significant scale factors for the S-MNL model. Finally, a taste-adjusted S-MNL model was used to estimate population-based preference weights (described below). Table 2 describes the five-step modelling approach, and Appendix 1 describes the model specifications.

The models were estimated by maximum likelihood using the BIOGEME [49]. ‘Apply runs’ were conducted to detect significant variables capturing taste heterogeneity, using the ALOGIT [50]. Every model used level_4 of the CONT attribute, ‘cont4’, ‘I have no control over my daily life’ as a reference attribute level. The constant and position coefficients of the first attribute in the choice set for the best and worst choices were also assigned a value of zero to prevent over-identification.^2^ We applied sandwich estimators to get robust standard errors, accounting for the repeated choices [51].

### Scale factors and learning and fatigue effects

To investigate scale factors, we included age, gender, education, SAH, overall QoL, experience in care, residential area, housing tenure, time to finalise eight BWS tasks and best and worst choices into the taste-and-scale MNL model (Table 2). Some of these factors were tested in Netten et al. [16]. We conducted a series of scale heterogeneity analyses with different subgroups of each variable for several sets of 4 or 5 potential scale variables to compare scale parameters and select scale variables. The final scale factors that were selected based on statistical significance (*p* < 0.05) were used in the S-MNL and taste-adjusted S-MNL models (Table 2).

The repeated and sequential choice tasks in choice experiments can cause fatigue and learning, affecting respondents’ choice behaviour [34–36]. We expected that the position of a choice task in a sequence of eight BWS choice tasks would be a scale factor explaining the error variance of the model. Following Carlsson et al. [34], we defined two identical sequences of four choice tasks in the BWS experiment. We tested the presence of fatigue or learning in the second sequence of four BWS choice tasks relative to the first sequence of four BWS choice tasks. Fatigue [learning] would mean that the respondents’ choice behaviour is less consistent [more consistent] in the last four BWS tasks than in the first four BWS tasks. Correspondingly, for fatigue [learning] to occur, the variance of the error term of the S-MNL model is higher [lower] in the last four tasks than in the first four tasks [34, 52].

### Final preference estimates

The preference weights should reflect the values of the Finnish general adult population. However, some socioeconomic and demographic covariates in the analysis sample were found to be over- or underrepresented compared to the general adult population (>10 percentage points of *p* < 0.05). This occurred in three subgroups: house/apartment renters (from housing tenure), those with lower secondary education or below (from education), and those without any religion (from religion) (Table 3). The taste-adjusted S-MNL model—i.e. an S-MNL model that included significant interaction terms between attribute levels and the subgroups above—was estimated, from which the attribute level coefficients were adjusted for significant taste differences between the sample and general populations using modified post-stratification [53] to derive the final preference weights. This correction method was also applied in previous studies [16, 19, 30, 54, 55]. The standard errors of the adjusted, population-weighted preference weights were calculated using fixed population weights (Table 3) and the estimated variance-covariance matrix of the parameters of Model [III] provided by BIOGEME [49].

**Table 3:** Analysis data characteristics vs. general population characteristics

Variable	Analysis data (*n* = 1005)		General adult population		Source
	%	Freq.	%	Freq.	
Socio-demographic variables					
			100	4,431,392	Statistics Finland (2016a)
Female	51.1	514	51.2	2,267,547	
Age (in years)			100	4,431,392	Statistics Finland (2016a)
18–24	9.3	93	10.3	455,977	
25–34	15.7	158	15.9	704,402	
35–44	15.2	153	15.1	671,350	
45–54	18.3	184	16.1	712,553	
55–64	25.5	256	16.6	737,135	
65–79	15.4	155	19.4	861,876	
80 or older	0.6	6	6.5	288,099	
Marital status			100	4,431,392	Statistics Finland (2016a)
Married	38.8	390	45.1	1,998,678	
Divorced	16.8	169	12.8	568,184	
Widowed	3.3	33	6.4	282,794	
Single	37.6	378	35.7	1,581,736	
Prefer not to say	3.5	35	–	–	
Education (ISCED 2011)			100	4,591,285	Statistics Finland (2015a)†
Lower secondary or below (≤ 2)	10.8	109	18.8	667,598	
Upper secondary (3, 4)	48.5	487	46.5	1,651,087	
Short-cycle tertiary (5)	10.4	105	10.5	373,847	
Bachelor’s or equivalent (6)	17.0	171	12.5	445,771	
Master’s or equivalent (7)	11.6	117	10.5	372,623	
Doctoral or equivalent (8)	1.6	16	1.2	42,449	
Employment status			100	4,431,392	Statistics Finland (2016b)
Self-employed persons	5.9	59	5.3	233,911	
Employees	36.2	364	45.8	2,022,548	
Students	7.4	74	5.4	240,405	
Pensioners	26.8	269	31.0	1,367,951	
Unemployed	15.9	160	8.5	374,534	
Others^#^	7.9	79	4.0	174,899	
Region			100	4,407,913	Statistics Finland (2016a)^†^
Helsinki and Uusimaa	25.1	252	29.7	1,311,203	
Southern Finland	30.0	301	21.5	948,790	
Western Finland	21.2	213	25.2	1,110,490	
North-Eastern Finland	23.8	239	23.5	1,037,430	
Religion			100	4,609,119	Statistics Finland (2016c)^†^
No religion	37.9	381	26.7	1,232,330	
Any religion	62.1	624	73.3	3,376,789	
Housing tenure			100	5,363,637	Statistics Finland (2015b)†
Own house or apartment	53.4	537	70.8	3,804,549	
Rent	46.4	466	27.4	1,471,006	
Other	0.2	2	1.9	101,544	

Variable	Analysis data (*n* = 1005)		General adult population		Source
	%	Freq.	%	Freq.	
Annual household disposable cash income					
≤ 16,440€ (1st or 2nd decile)	29.6	297			
16,441€–26,230€ (3rd or 4th decile)	18.6	187			
26,231€–38,010€ (5th or 6th decile)	15.5	156			
38,011€–54,690€ (7th or 8th decile)	13.6	137			
≥ 54,691€ (9th or 10th decile)	10.9	110			
Prefer not to say or do not know	11.7	118			
Health and well-being					
Overall quality of life (QoL)					Murto et al. [67]
So good or very good	19.3	194	22.7		Very good
Good	47.9	481	54.9		Good
Alright	22.2	223	18.3		Neither good nor poor
Bad	8.4	84	3.5		Poor
Very bad or so bad	2.3	23	0.7		Very poor
Self-assessed health (SAH)					Murto et al. [67]
Very good	7.9	79	29.9		Good
Good	46.1	463	36.2		Quite good
Fair	35.6	358	24.6		Fair
Bad	9.7	97	7.9		Quite bad
Very bad	0.8	8	1.5		Bad
Experience of caring and need for social care					
(i) Have you (personally) provided help or support to anyone in the last month because they have long-term physical or mental ill-health or disability, or problems relating to old age?					
Yes	36.8	370			
No	63.2	635			
(ii) Have either you or someone you are close to ever been in need of any regular help and long-term care over the last 10 years?					
Yes, I have or my partner/one of my parents has personal experience	36.2	364			
Yes, one of my children/siblings or another relative/friend or an acquaintance or a colleague or a neighbour	23.8	239			
No experience with long-term care needs or do not know^&^	40.0	402			
Understanding the tasks					
(i) Did you feel that you could put yourself in the imaginary situations described in the best-worst exercises?					
Yes, all of the time	57.9	582			
Yes, but only some of the time	38.7	389			
No	3.4	34			
(ii) In the best-worst exercises, did you understand the situations?					
Yes, all of them	81.7	821			
Yes, but only some of them	17.0	171			
No	1.3	13			

^#^Those who were permanently sick or disabled, in community or military services, doing housework or outside of labour force

^†^Religion (Statistics Finland 2016c) and education (Statistics Finland 2015a) refer to the population aged 15 or older. Housing tenure (Statistics Finland 2015b) refers to the whole housing population. Regions (Statistics Finland 2016a) refer to the population aged 18 or older. Household disposable cash income excluded imputed rents

^&^Including 4% of respondents who replied to response item “don’t know”

We normalised the attribute-level coefficients from different estimated models using the largest attribute-level coefficient as the common denominator. To better understand quantified changes in different ASCOT-QoL states, we linearly transformed the final 28 preference estimates to an index. We anchored the ASCOT-Carer index at a value of one for the set of states presented by the seven highest attribute-level coefficients (each per attribute) and a value of zero for the set of states presented by the seven lowest attribute-level coefficients (each per attribute), keeping the relative differences between the attribute-level coefficients unchanged. Thus, the ASCOT-Carer index measuring carers’ SCRQoL ranges between zero and one, where one represents the best SCRQoL represented by the seven best ASCOT-QoL states (each per attribute) and zero represents the worst SCRQoL represented by the seven worst ASCOT-QoL states (each per attribute). The transformation method has been used to anchor country-specific preference weights [19, 30, 42, 55, 56].

## Results

### Sample characteristics

Compared to the general adult population, the analysis sample had more people aged 55–64 years, fewer who were employed, fewer people with the lowest educational level, a higher proportion of people with no religion (i.e. fewer people with some religion) and fewer homeowners (Table 3). 36.8% of respondents reported that they personally provided help or support to someone with long-term physical or mental ill-health or disability in the last month. Concerning how often respondents were able to put themselves in the imaginary situations described in the BWS exercises, 57.9% of them were able to do so all the time and 38.7% some of the time. Almost every respondent reported that they had understood the situations in the best-worst exercises all or some of the time (98.7%) (Table 3).

The *cont1*, *occu2*, *occu1* and *spac1* attribute levels were mostly selected as the best or second-best (best, for simplicity’s sake) choices (Table 4). The *cont4*,* occu4*,* spac4* and *safe4* attribute levels were mostly chosen as the worst or second-worst (worst, for simplicity’s sake) choices. The *perc2* attribute level was preferred to the *perc1* attribute level;* perc2* was selected more often than *perc1* as the best or worst choice and in total. For the best choices, the further away from the 1st position in the profile an attribute level is, the less likely it was selected. For the worst choices, the likelihood of selecting an attribute level increased from the 1st to the 7th position, but respondents seemed to be indifferent to the 3rd or 4th positions in the profile (Table 4).

**Table 4:** Descriptive statistics of attribute, attribute levels and position variables in the BWS tasks (*n* = 32,160)

		Descriptive value		
	Name	Mean		
		All	Best/2nd-best choice	Worst/2nd-worst choice
Attribute and level				
Occupation	OCCU	0.165	0.195	0.135
1. I am able to spend my time as I want, doing things I value or enjoy.	occu1	0.045	0.085	0.005
2. I am able do enough of the things I value or enjoy with my time.	occu2	0.045	0.085	0.005
3. I do some of the things I value or enjoy with my time, but not enough.	occu3	0.027	0.020	0.034
4. I do not do anything I value or enjoy with my time.	occu4	0.047	0.004	0.090
Control over daily life	CONT	0.173	0.197	0.149
1. I have as much control over my daily life as I want.	cont1	0.048	0.090	0.006
2. I have adequate control over my daily life.	cont2	0.043	0.080	0.005
3. I have some control over my daily life, but not enough.	cont3	0.030	0.023	0.037
4. I have no control over my daily life.	cont4	0.052	0.004	0.100
Looking after yourself	PERC	0.135	0.122	0.148
1. I look after myself as well as I want.	perc1	0.029	0.053	0.005
2. I look after myself well enough.	perc2	0.030	0.055	0.006
3. Sometimes I cannot look after myself well enough.	perc3	0.034	0.008	0.060
4. I feel I am neglecting myself.	perc4	0.041	0.006	0.077
Safety	SAFE	0.126	0.068	0.184
1. I feel as safe as I want.	safe1	0.029	0.050	0.008
2. Generally I feel adequately safe, but not as safe as I would like.	safe2	0.018	0.011	0.026
3. I feel less than adequately safe.	safe3	0.036	0.004	0.068
4. I do not feel at all safe.	safe4	0.043	0.003	0.082
Social participation and involvement	SOCI	0.121	0.111	0.131
1. I have as much social contact as I want with people I like.	soci1	0.030	0.053	0.007
2. I have adequate social contact with people.	soci2	0.024	0.037	0.011
3. I have some social contact with people, but not enough.	soci3	0.027	0.017	0.037
4. I have little social contact with people and feel socially isolated.	soci4	0.040	0.004	0.076
Space and time to be yourself	SPAC	0.168	0.190	0.147
1. I have all the space and time I need to be myself.	spac1	0.044	0.084	0.005
2. I have adequate space and time to be myself.	spac2	0.041	0.075	0.007
3. I have some of the space and time I need to be myself, but not enough.	spac3	0.036	0.026	0.045
4. I do not have any space or time to be myself.	spac4	0.047	0.005	0.090
Feeling supported and encouraged	SUPP	0.112	0.118	0.107
1. I feel I have the encouragement and support I want.	supp1	0.027	0.049	0.005
2. I feel I have adequate encouragement and support.	supp2	0.031	0.051	0.011
3. I feel I have some encouragement and support, but not enough.	supp3	0.018	0.012	0.024
4. I feel I have no encouragement and support.	supp4	0.037	0.006	0.067

Attribute position	Name	Mean	Std. dev
For best/second-best choices			
Attribute appeared in the 1st row	pos1_B	0.080	0.271
Attribute appeared in the 2nd row	pos2_B	0.078	0.268
Attribute appeared in the 3rd row	pos3_B	0.075	0.263
Attribute appeared in the 4th row	pos4_B	0.073	0.259
Attribute appeared in the 5th row	pos5_B	0.069	0.254
Attribute appeared in the 6th row	pos6_B	0.063	0.243
Attribute appeared in the 7th row	pos7_B	0.062	0.242
For worst/second-worst choices			
Attribute appeared in the 1st row	pos1_W	0.069	0.253
Attribute appeared in the 2nd row	pos2_W	0.071	0.256
Attribute appeared in the 3rd row	pos3_W	0.070	0.255
Attribute appeared in the 4th row	pos4_W	0.070	0.254
Attribute appeared in the 5th row	pos5_W	0.073	0.260
Attribute appeared in the 6th row	pos6_W	0.075	0.263
Attribute appeared in the 7th row	pos7_W	0.074	0.262
Scale variable			
= 1 (good health) if the participant reported a very good or good health state, and 0 otherwise (i.e. fair or bad or very bad health state)	hgood	0.539	0.498
= 1 (long time) if the participant used more than 6.5 min to complete the BWS tasks, and 0 otherwise (i.e. short time)^#^	tmlong	0.750	0.433
= 1 (high education) if the participant had a Bachelor or Master or Doctoral or equivalent degree, and 0 otherwise (i.e. low education, if she/he had a short-cycle tertiary education or lower education)	eduhigh	0.302	0.459
= 1 (learning) if being in the second sequence of four BWS tasks, 0 otherwise (i.e. the first sequence of four BWS tasks)	learning	0.500	0.500

©University of Kent: the ASCOT-Carer measure is reproduced with permission from the University of Kent. All rights reserved

^#^Time to complete the BWS task: (p25; p50; p75; mean) = (6.5; 8.7; 12.1; 23.4) min

### The preference estimates

Results from the basic MNL (Model [I]) and S-MNL (Model [II]) and taste-adjusted S-MNL (Model [III*]) are reported in Table 5. In Model [III*], the coefficients of the *occu3*, *safe4*, *soci1* and *supp4* attribute levels were adjusted to the significant taste differences between the sample and the general populations, all other estimated coefficients being the same as in Model [III] (Supplemental Table S1). Since pseudo-R^2^ with values in the [0.3; 0.4] range correspond to an *R*^2^ with values in the [0.6; 0.8] range for an equivalent linear regression [57], the pseudo-*R*^2^ of 0.289 presents a decent fit for Model [III*].^3^

**Table 5:** Estimated preference parameters for the Finnish ASCOT for carers (*n* = 32,160)

	Model [I]^1^			Model [II]^1^			Model [III*]^1,2^		
	Estimated	Robust	Normalised	Estimated	Robust	Normalised	Estimated	Robust	Normalised
	Coeff.	*t*-value	Coeff.	Coeff.	*t*-value	Coeff.	Coeff.	*t*-value	Coeff.
Occupation (OCCU)									
occu1	4.617	37.18	0.973	3.351	15.46	0.976	3.353	15.46	0.976
occu2	4.582	37.76	0.965	3.333	15.66	0.970	3.336	15.65	0.971
occu3	2.211	31.54	0.466	1.597	14.76	0.465	1.592	14.46	0.463
occu4	0.433	8.75	0.091	0.302	7.67	0.088	0.303	7.67	0.088
Control over daily life (CONT)									
cont1	4.746	36.16	1.000	3.435	15.32	1.000	3.437	15.31	1.000
cont2	4.439	37.34	0.935	3.232	15.58	0.941	3.235	15.57	0.941
cont3	2.227	28.68	0.469	1.603	14.39	0.467	1.604	14.38	0.467
cont4	0.000	ref.	0.000	0.000	ref.	0.000	0.000	ref.	0.000
Looking after yourself (PERC)									
perc1	3.773	36.72	0.795	2.756	15.67	0.802	2.758	15.67	0.802
perc2	3.813	36.05	0.803	2.782	15.58	0.810	2.784	15.58	0.810
perc3	1.290	20.37	0.272	0.935	13.01	0.272	0.936	13.01	0.272
perc4	0.889	15.13	0.187	0.634	11.18	0.185	0.635	11.17	0.185
Safety (SAFE)									
safe1	3.642	36.71	0.767	2.647	15.55	0.771	2.648	15.55	0.770
safe2	2.336	32.80	0.492	1.683	15.00	0.490	1.684	15.00	0.490
safe3	1.138	18.85	0.240	0.810	12.16	0.236	0.811	12.16	0.236
safe4	0.778	13.17	0.164	0.563	10.39	0.164	0.608	10.29	0.177
Social participation and involvement (SOCI)									
soci1	3.766	34.82	0.794	2.741	15.42	0.798	2.758	15.20	0.803
soci2	3.344	33.89	0.705	2.439	15.51	0.710	2.440	15.50	0.710
soci3	2.203	31.03	0.464	1.584	14.83	0.461	1.586	14.82	0.461
soci4	0.935	16.46	0.197	0.674	11.70	0.196	0.674	11.69	0.196
Space and time to be yourself (SPAC)									
spac1	4.579	37.82	0.965	3.326	15.69	0.968	3.328	15.69	0.968
spac2	4.307	37.27	0.908	3.149	15.71	0.917	3.151	15.70	0.917
spac3	2.118	29.50	0.446	1.528	14.62	0.445	1.530	14.61	0.445
spac4	0.397	7.84	0.084	0.287	7.21	0.084	0.287	7.20	0.083
Feeling supported and encouraged (SUPP)									
supp1	3.662	36.37	0.772	2.665	15.55	0.776	2.667	15.54	0.776
supp2	3.660	35.08	0.771	2.662	15.42	0.775	2.664	15.41	0.775
supp3	2.228	30.92	0.469	1.611	15.06	0.469	1.612	15.06	0.469
supp4	1.097	18.36	0.231	0.779	12.27	0.227	0.798	12.19	0.232
Position for best/second-best choices									
pos2_B	− 0.050	− 1.18		− 0.029	− 0.95		− 0.029	− 0.96	
pos3_B	− 0.148	− 3.34		− 0.109	− 3.38		− 0.109	− 3.38	
pos4_B	− 0.220	− 4.98		− 0.151	− 4.67		− 0.151	− 4.67	
pos5_B	− 0.276	− 5.86		− 0.194	− 5.49		− 0.194	− 5.49	
pos6_B	− 0.414	− 9.18		− 0.285	− 7.78		− 0.285	− 7.78	
pos7_B	− 0.412	− 8.79		− 0.288	− 7.82		− 0.288	− 7.82	
Position for worst/second-worst choices									
pos2_W	− 0.047	− 1.07		− 0.036	− 1.12		− 0.036	− 1.13	
pos3_W	− 0.017	− 0.38		− 0.008	− 0.24		− 0.007	− 0.22	
pos4_W	0.041	0.93		0.035	1.08		0.034	1.07	
pos5_W	− 0.058	− 1.27		− 0.039	− 1.19		− 0.041	− 1.24	
pos6_W	− 0.064	− 1.37		− 0.042	− 1.25		− 0.043	− 1.29	
pos7_W	− 0.034	− 0.74		− 0.025	− 0.74		− 0.026	− 0.77	
Scale factor									
hgood				1.120	2.13		1.120	2.13	
eduhigh				1.244	3.69		1.243	3.63	
tmlong				1.241	3.23		1.240	3.21	
learning				1.069	3.88		1.069	3.93	
Observations		32,160			32,160			32,160	
Degree of freedom		39			43			47	
Log-likelihood value		− 38,685.26			− 38,475.50			− 38,469.54	
Rho^2^ (0)		0.285			0.288			0.289	

^1^Model [I] = basic MNL. Model [II] = S-MNL. Final preference weights reported in Model [III*] were derived using results from Model [III] (taste-adjusted S-MNL) (Supplemental Table S1)

^2^We corrected the coefficients of occu3, safe4, soci1, and supp4 attribute levels and computed their robust *t*-values

The inclusion of four scale factors substantially improved the goodness-of-fit of the model. The log-likelihood value increased from −38,685.26 (Model [I]) to −38,475.50 (Model [II]). The large increase in the log-likelihood value of Model [II] compared to Model [I] implied that it is very important to account for scale heterogeneity. Although the attribute-level coefficients from Models [II] and [Model III*] were quite similar, the latter was significantly better than the former by the log-likelihood ratio test {LR statistic 11.92 = –2 × (–38,457.50–(–38,469.54)); df = 47–43 = 4; *p* = 0.018} (Table 5). Below, we focus on the results from Model [III*] if not otherwise specified.

**Fig. 2:**
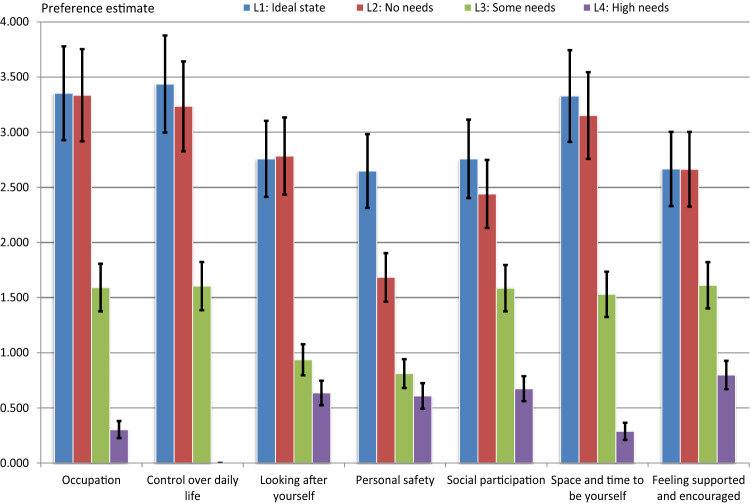
The attribute-level coefficients and their 95% confidence interval for the Finnish ASCOT for carers measure

Across all attributes, the estimated attribute-level coefficients indicating their importance relative to *cont4* were statistically significant. The three most-valued attribute levels were found within the control over daily life, occupation and space-and-time attributes (Fig. 2). The *cont1* attribute level was the most-valued ASCOT-QoL state (coefficient 3.437). This was followed by the *occu1* (3.343) and *occu2* (3.336) attribute levels and the *spac1* (3.328) attribute level (Table 5). Furthermore, within each attribute, the bottom level (level_4) was the least-valued state. The least-valued attribute level, *cont4*, was followed by the *spac4* (coefficient 0.287) and *occu4* (0.303) attribute levels. The next three smallest valued states were the *safe4* (coefficient 0.608), *perc4* (0.635) and *soci4* (0.674) attribute levels.

Based on the magnitudes of the coefficients, the two top attribute levels were appreciated more than the two bottom attribute levels. Except for the SAFE attribute, the difference between attribute levels 1 and 2 was not significant at a 5% level of significance. In addition, a higher value was placed on the difference between attribute levels 2 and 3 (i.e. moving up from level_3 [some needs] to level_2 [no needs]) than on the difference between attribute levels 1 and 2 (i.e. moving up from level_2 to level_1 [ideal state]) and a higher value was also placed on the difference between attribute levels 3 and 4 than on the difference between attribute levels 1 and 2. Apart from the PERC attribute, the ordering of the attribute levels described by the magnitude of their estimated coefficients followed the ordering of four levels defined for each ASCOT-Carer attribute (Table 5, Fig. 2).

The result that the coefficient of the *perc2* attribute level was greater than that of the *perc1* attribute level was unexpected. Due to this, we ran a new taste-adjusted S-MNL with the restriction that these coefficients were the same. This restriction did not have much influence on the estimated coefficients of the other attribute levels, while the new joint coefficient for *perc1* and *perc2* (Model [IV]) was the exact average of the coefficients of *perc1* and *perc2* (Model [III]) (Supplemental Table S1). Compared to the unrestricted model [III], the restricted model [IV] was also supported by the likelihood ratio test (LR statistic = 0.60; df = 1; *p* = 0.436). However, to keep the order of the ASCOT attribute levels indicating the need intensity and ease the application of the preference weights, we used the preference estimates reported in Model [III*] (Table 5), from which we switched the order of the estimated coefficients of the *perc1* and *perc2* attribute levels for the final preference weights to be used in practice (Table 6).

**Table 6:** Values of the Finnish preference weights for the ASCOT for carers’ measure

Value of preference weight	Level and meaning	Occupation	Control over daily life	Looking after yourself	Personal safety	Social participation	Space and time	Feeling supported and encouraged
Panel 1. Normalised values	1 Ideal state	0.976	1.000	0.810	0.770	0.803	0.968	0.776
	2 No needs	0.971	0.941	0.802	0.490	0.710	0.917	0.775
	3 Some needs	0.463	0.467	0.272	0.236	0.461	0.445	0.469
	4 High needs	0.088	0.000	0.185	0.177	0.196	0.083	0.232
Panel 2. Preference-based index values	1 Ideal state	0.163	0.168	0.131	0.123	0.129	0.162	0.124
	2 No needs	0.162	0.156	0.129	0.069	0.111	0.152	0.124
	3 Some needs	0.063	0.064	0.026	0.019	0.063	0.060	0.065
	4 High needs	− 0.010	− 0.027	0.009	0.008	0.011	− 0.011	0.018

*Note*. For the looking after yourself attribute, the current preference weight of level_1 was the originally estimated preference weight of level_2 and the current preference weight of level_2 was the originally estimated preference weight of level_1

Significant position effects were found for the best choices. Compared to the top position of the presentation of the attributes, the coefficient of the second position variable (pos2_B) did not differ statistically significantly from that of the first position (*p* > 0.05). However, the coefficients of the position variables other than pos2_B were all statistically significant (Table 5). Moreover, the negative signs of the coefficients indicate that the respondents were less likely to choose an item in the profile that appeared after the second item from the top.

For the worst choices, the coefficients of the position variables were not statistically significant. The negative coefficients imply that the respondents were less likely to choose items located in the 6th and 5th rows of the profile than the items on the top or bottom rows when making their worst choices. Furthermore, except for pos2_W and pos2_B, the coefficients of the position variables were of lower magnitude for the worst choices than for the best choices. The results imply that the position effect was more strongly related to the best choices than to the worst choices, other things remaining constant, which was in agreement with the result from a discrete choice experiment [58].

### The scale factors and learning effect

The estimated parameter for the learning scale factor exceeds 1 (Table 5). We thus found a lower error variance for the second sequence of four tasks relative to the first sequence of four tasks, suggesting that the respondent responses were more consistent in the last four tasks than in the first four tasks, i.e. that learning took place in the sequential BWS choice experiment. Our finding is consistent with that by Carlsson et al. [33], who explored learning and fatigue effects in the context of a choice experiment concerning food safety.

Regarding other scale factors, respondents who had better SAH, or a high level of education or spent more time (> 6.5 min) doing the BWS tasks were more consistent in their choices than those who had worse (i.e. fair, bad or very bad) SAH, or a lower level of education or spent less time (≤ 6.5 min) doing the BWS tasks (Table 5). The latter two scale factors were in line with the results in Batchelder et al. [19].

### The final preference weights

Table 6 reports the normalised and rescaled values (i.e. preference-based index values) of the attribute-level coefficients for the Finnish ASCOT-Carer measure. Due to differences between the attribute-level coefficients and the average value of all lowest rated attribute levels [42, 55], the rescaled values can also be negative. The originally estimated coefficients of the *perc1* and *perc2* attribute levels were switched, as discussed above (Table 6).

**Fig. 3:**
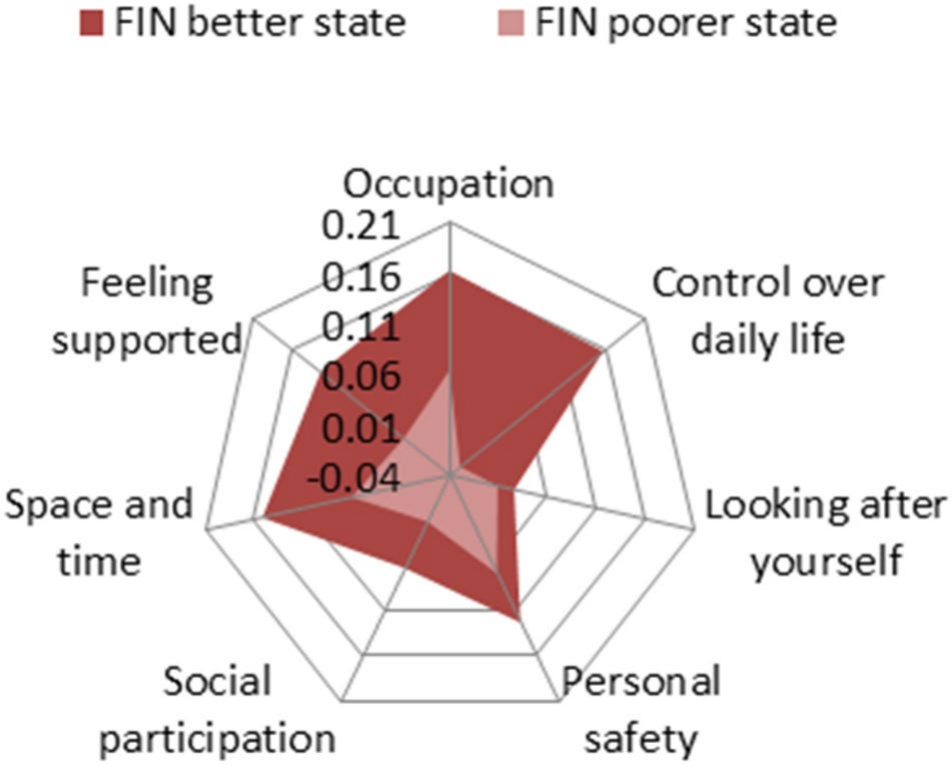
Changes in the Finnish preference-based index values for the ASCOT-Carer measure from a poorer state (3442434) to a better state (1231321). Preference-based index values for the Finnish version of the ASCOT-Carer measure were derived in this study (Table 6). The state of 3442434 consisted of occu3, cont4, perc4, safe2, soci4, space3, and supp4 attribute levels and that of 1231321 consisted of occu1, cont2, perc3, safe1, soci3, space2, and supp1 attribute levels

Preference-based index values for the Finnish ASCOT-Carer measure can be used to measure changes in carers’ SCRQoL, for instance, due to targeted interventions aiming to improve carers’ QoL (Table 6). Since the ASCOT-Carer index is additive, an improvement in the ASCOT-QoL of an individual—for example, from an inferior state of 3442434 to an improved state of 1231321—would suggest a change in value from 0.204 [= 0.063 + (−0.027) + 0.009 + 0.069 + 0.011 + 0.069 + 0.018] to 0.808 [= 0.163 + 0.156 + 0.026 + 0.123 + 0.063 + 0.152 + 0.124].^4^ This gain in SCRQoL is illustrated as the area between two acreages covered by two radars in Fig. 3. Although with a different scale, a similar figure can be drawn using the normalised values. Those who would like to utilise our developed preference weights can use the normalised or rescaled values of the final preference weights in evaluations involving the Finnish ASCOT-Carer measure (Table 6).

## Discussion

In this study, we derived the population-based preference weights for the Finnish version of the ASCOT-Carer measure, which was translated from the English ASCOT-Carer measure [18] to Finnish in 2015–2016 [31]. The BWS choice data were analysed using an S-MNL model, considering the significant taste differences between the sample and general adult populations. Moreover, we provided evidence on the learning effect in the BWS experiment.

Both the most and least-valued attribute levels of the Finnish ASCOT-Carer measure were found in the *occupation*, *control* and *space-and-time* attributes. Compared to English preference weights that were derived using a similar analysis framework [19], Finnish respondents valued most highly the attribute levels within the *control*, *occupation* and *space-and-time* attributes (Supplemental Figure O1). The most preferred attribute level was *cont1* state in Finland, while it was *occu1* in England. For both countries, the least preferred attribute level was the *cont4* state with a negative preference-based index value: − 0.027 (Finland) and − 0.012 (England). Although the rank order of the derived preference weights is similar between the two countries, there are clear differences in the magnitude of the country-specific preference weights, which could stem from differences in the country-specific populations’ preferences and values or norms. These differences justify the contribution of this paper to developing the Finnish preference weights for the Finnish ASCOT-Carer measure.

The significant position effect we found for the best choices was in line with the English [19] and Austrian [30] studies. To mitigate position bias affecting choice behaviour and decision rules, which can result in invalid coefficient estimates, the position of the attributes in the BWS profiles should be rotated to ensure that each item will appear an equal number of times in each profile. This was earlier noted by Campbell and Erdem [58]. Since the position effect can bring about invalid preference estimates [34], in addition to randomisation at the experimental design stage, researchers can include position-specific constants of the attributes into the model to account for the order of the profiles.

The significant scale factors found in this study suggest that researchers should account for scale heterogeneity because varying error variance across different sample population groups can distort preference estimates [47]. This also calls for approaches that can disentangle scale heterogeneity from taste heterogeneity to make accurate estimates about people’s preferences [59]. This, in turn, supports our approach of investigating taste heterogeneity first (using the mixed logit with observed characteristics of respondents) and then scale heterogeneity after having controlled for taste heterogeneity (using the G-MNL) before obtaining the final preference estimates from the S-MNL model.

Education and health as scale factors are known to be related to cognitive functioning [42, 60]. Besides implying the use of heuristics to quickly make choices [61], short response times can imply respondents’ reduced effort to engage in the BWS tasks or to properly consider the available alternatives. The evidence of the learning effect in the sequential BWS choice experiment is consistent with the previous choice experimental studies [52, 62]. As we had two identical sequences of four BWS tasks, the finding implies the more consistent responses in the second half of the experiment than in the first half. We also tested other sequential divisions of the BWS choice tasks as a scale factor (such as 1 task vs. 7 tasks; 2 tasks vs. 6 tasks; 3 tasks vs. 5 tasks), but they were not statistically significant. The learning effect implies that future studies that collect and use sequential choice data should develop study designs that can reduce possible preference uncertainty at the beginning of the experiment and increase respondent engagement throughout the experiment. Concerning scale heterogeneity, researchers can account for the effect of learning and fatigue on the preference estimates by explicitly modelling learning or fatigue as a scale factor using the sequences of the BWS tasks.

There is evidence that modes of survey administration, such as Internet-based surveys, might result in stronger fatigue effects and weaker learning effects [36]. Although the survey including the BWS experiment was Internet based, we found the learning effect. Prominent differences in the preferences for SCRQoL from two models, which used online BWS data and face-to-face interview data, were not observed [63]. The final pattern of learning and fatigue as a research question is beyond the scope of this study. However, it might be relevant to investigate in more detail the potential impact of the learning effects on preference stability and how learning styles and preference uncertainty vary between the individuals [34]. The found learning effect would suggest that these issues could extend to also consider the BWS method in different survey administration modes.

This study contributes to expanding the number of valid measures that can be used to evaluate capability-based QoL in a general population [37] and to consider the evaluation of outcomes and interventions beyond health [64, 65]. Since the ASCOT [16] focuses on measuring care recipients’ SCRQoL and the ASCOT-Carer [19] focuses on measuring caregivers’ SCRQoL, both measures can be in use for the evaluation of social care interventions. Finnish preference weights for the ASCOT measure have been established [29].

Our study has some limitations. Despite the use of sampling quotas, the online panel was not fully representative regarding housing tenure, education and religion. However, we adjusted the preference weights to better reflect the values of the Finnish general adult population, which was done in the studies [19, 30], but in addition, we computed the standard errors of the adjusted final preference estimates, which was not carried out in the mentioned studies. With the used survey administration method, we were not able to monitor external and internal incentives or impetuses during the BWS experiment, such as the respondents’ behaviour, engagement and burden, and changes in the task environment. Nevertheless, respondents who spent less than 4.5 min doing the BWS tasks were excluded during the data collection phase.

We have successfully derived the Finnish preference weights for the Finnish ASCOT-Carer measure. The preference weights established here will enable researchers in Finland, for the first time, to consider the value of different social care interventions for evaluating support and services to informal carers. The learning effect, as one of the significant scale factors, implies not only the importance of accounting for scale heterogeneity in the choice experiments but also that future studies with sequential choice tasks should develop study designs such that they ensure equal consideration to all choice tasks (or profiles) for the attributes in the profiles to reduce potential preference uncertainty at the beginning of the experiment and increase respondent engagement in the experiment.

### Supplementary Information

Below is the link to the electronic supplementary material.

Supplementary file1 (DOCX 46 KB)

## Data Availability

Data are confidential and are not available for public use.

## Appendix 1

### Model specifications

Based on random utility theory, the utility associated with chosen attribute* j* that individual* i* gets,* U*_ij_, is given by an explained (systematic) component,* V*_ij_, and a random (error) component, $\xi_{ij}$ [48]:

1 $$U_{ij} = V_{ij} + \left( {{{\xi_{ij} } \mathord{\left/ {\vphantom {{\xi_{ij} } \lambda }} \right. \kern-\nulldelimiterspace} \lambda }} \right).$$

The scale parameter *λ* is inversely proportional to the standard error of the error term, allowing the variance of the error term to vary across different subpopulations in the data. The higher the value of *λ*, the lower the error variance of the random utility model is, implying more consistent choices. However, in the basic MNL model, *λ* is set to unity. In addition, the explained component was modelled as follows:

2 $$V_{ij} = \sum\limits_{p = 1}^{7} {\alpha_{p}^{b} } z^{b} + \sum\limits_{p = 1}^{7} {\alpha_{p}^{w} } z^{w} + \sum\limits_{p = 1}^{7} {\sum\limits_{q = 1}^{4} {\beta_{pq} x_{pq} } } ,$$

where *x* stands for an attribute level that was independently and sequentially selected, β_pq_ represents the effect of the q*th* level (1 ideal state, 2 no needs, 3 some needs, 4 high needs) over the p*th* attribute (1 occupation, 2 control over daily life, 3 looking after yourself, 4 safety, 5 social participation and involvement, 6 space and time to be yourself, 7 feeling supported and encouraged). z^b^ [or z^w^] stands for dummy variables that were associated with the position (or order) of the attribute chosen as the best or second-best [or worst or second-worst] within the choice set; $\alpha_{p}^{b}$ ${\text{[or }}\alpha_{p}^{w} ]$ is the position (or ordering) effect of the p*th* attribute for the best or second-best [or worst or second-worst] choices.

Assuming that the error term in model (1) is independently and identically type I extreme-value distributed, the probability that an individual *i* chooses alternative *j* from all the possible alternatives *s* in a choice set *S* is given as [51]:

3 $$P_{ij} = \frac{{{\text{exp}}(\lambda V_{ij} )}}{{\sum\nolimits_{s \in S} {{\text{exp}}(\lambda V_{is} )} }}.$$

To simultaneously analyse “worst” and “best” choice data, the utility of the “worst” or “second-worst” (hereafter “worst”) choice has a sign opposite to the sign of the utility associated with the “best” or “second-best” (hereafter “best”) choice, while the utility functions for both types of choices are similar [40]. In each estimated model, we included as basic explanatory variables the attribute levels and variables describing attribute-positions for the best and worst choices. The latter allowed for the overall effects of attribute ordering associated with the experimental task design [58, 66]. Owing to scale factors, the number of parameters estimated by an S-MNL will be higher than that by a basic MNL.

## Footnotes

1 Both four-level self-completion (SCT4) and interview (INT4) questionnaires for both the ASCOT and ASCOT-Carer were translated into Finnish (www.pssru.ac.uk/ascot/translations).

2 We included attribute-specific constants for the worst or second-worst choices into the mixed logit and G-MNL models. To avoid a saturated model, one attribute-specific constant was used as the reference constant.

3 Rho^2^ (0) produced by BIOGEME [49] is pseudo-R^2^.

4 The attribute levels indicated the intensity of needs: 1 = ideal state; 2 = no needs; 3 = some needs; and 4 = high needs. The attributes were specified in the order: 1. Occupation (OCCU), 2. Control over daily life (CONT), 3. Looking after yourself (PERC), 4. Personal safety (SAFE), 5. Social participation (SOCI), 6. Space and time (SPAC), and 7. Feeling supported and encouraged (SUPP). The state of 3442434 consisted of *occu3*,* cont4*,* perc4*,* safe2*,* soci4*,* space3*, and *supp4* attribute levels and that of 13121 consisted of *occu1*,* cont2*,* perc3*,* safe1*,* soci3*,* space2*, and *supp1* attribute levels.
