# Supplementary material for: Valuing informal carers’ quality of life using best-worst scaling—Finnish preference weights for the Adult ﻿Social Care Outcomes Toolkit for carers (ASCOT-Carer)
Source: Eur J Health Econ. 2021 Sep 1;23(3):357–74. doi: 10.1007/s10198-021-01356-3 (PMC8964536; doi:10.1007/s10198-021-01356-3)
Supplement: Supplementary file 1 — Supplementary file1 (DOCX 46 KB) [file 10198_2021_1356_MOESM1_ESM.docx]

Supplemental Table S1. Estimation results from the unrestricted and restricted taste-adjusted S-MNL models for the Finnish ASCOT for carers measure (n = 32,160)

Supplemental Table S1. Estimation results from the unrestricted and restricted taste-adjusted S-MNL models for the Finnish ASCOT for carers measure (n = 32,160), *continued*

*Sources*. Batchelder et al. (2019) and this study (Nguyen et al., 2021).

Supplemental Figure O1. Comparison of Finnish and English preference-based index values for the ASCOT for carers measure
